# Finding Novel Molecular Connections between Developmental Processes and Disease

**DOI:** 10.1371/journal.pcbi.1003578

**Published:** 2014-05-29

**Authors:** Jisoo Park, Heather C. Wick, Daniel E. Kee, Keith Noto, Jill L. Maron, Donna K. Slonim

**Affiliations:** 1 Department of Computer Science, Tufts University, Medford, Massachussetts, United States of America; 2 Department of Pediatrics, The Floating Hospital for Children at Tufts Medical Center, Boston, Massachusetts, United States of America; 3 Department of Pathology, Tufts University School of Medicine, Boston, Massachusetts, United States of America; National Center for Biotechnology Information (NCBI), United States of America

## Abstract

Identifying molecular connections between developmental processes and disease can lead to new hypotheses about health risks at all stages of life. Here we introduce a new approach to identifying significant connections between gene sets and disease genes, and apply it to several gene sets related to human development. To overcome the limits of incomplete and imperfect information linking genes to disease, we pool genes within disease subtrees in the MeSH taxonomy, and we demonstrate that such pooling improves the power and accuracy of our approach. Significance is assessed through permutation. We created a web-based visualization tool to facilitate multi-scale exploration of this large collection of significant connections (http://gda.cs.tufts.edu/development). High-level analysis of the results reveals expected connections between tissue-specific developmental processes and diseases linked to those tissues, and widespread connections to developmental disorders and cancers. Yet interesting new hypotheses may be derived from examining the unexpected connections. We highlight and discuss the implications of three such connections, linking dementia with bone development, polycystic ovary syndrome with cardiovascular development, and retinopathy of prematurity with lung development. Our results provide additional evidence that 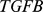 plays a key role in the early pathogenesis of polycystic ovary syndrome. Our evidence also suggests that the *VEGF* pathway and downstream *NFKB* signaling may explain the complex relationship between bronchopulmonary dysplasia and retinopathy of prematurity, and may form a bridge between two currently-competing hypotheses about the molecular origins of bronchopulmonary dysplasia. Further data exploration and similar queries about other gene sets may generate a variety of new information about the molecular relationships between additional diseases.

## Author Summary

Understanding the roles that genes involved in normal human development can play in disease processes is an important part of predicting disease risk and designing novel treatment approaches. In this study, we have identified classes of disease that are associated with a surprisingly large number of genes involved in any of several tissue-specific developmental processes. To do so, we developed a novel approach whose strength comes from pooling genetic information across related diseases, overcoming problems ordinarily posed by limited information about individual gene-disease relationships. We demonstrate the method's efficacy both by examining its ability to highlight connections between gene sets and disease classes that are known to be related, and by demonstrating that the approach recovers expected broad classes of connections, such as those between heart development and cardiovascular disorders. However, by examining unexpected connections in this data set, we are able to develop new understanding of some surprising disease relationships, such as the one between dementia and osteoporosis. Such connections may lead to a better overall understanding of the role of development in lifelong health, as well as to the design of new methods to treat a range of diseases.

## Introduction

The study of the health implications of developmental processes has now entered the genomic era. The recent sequencing of an entire fetal genome [1] has demonstrated the possibility of applying molecular methods to design novel prenatal diagnostics. The development of therapeutic approaches for personalized fetal treatment of developmental disorders is now on the horizon [2]. Genomic approaches are providing new insights into causes of and possible treatments for such widespread pediatric disorders as asthma [3] and autism [4]. A growing awareness that development may influence lifelong health risk [5], [6] has led to closer examination of the molecular links between developmental processes and disease at multiple life stages.

Despite considerable progress, our understanding of the molecular etiology of most complex diseases is still limited. Yet by combining weak signals from multiple genes, we may identify patterns that provide clinically significant insights into disease processes. We hypothesized that by examining the relationships between sets of genes related to specific developmental processes and reported disease genes, we could develop novel insights into developmental impacts on health. To test this hypothesis, we created a novel approach and tool to assess the overrepresentation of various developmental gene sets among groups of genes linked to specific diseases. Our approach derives its strength from combining signals of sets of genes and from pooling disease-gene links across disease subtypes using a hierarchical taxonomy of disease. We demonstrate that this pooling approach improves accuracy over a comparable enrichment-detection method without pooling. Our approach has the advantage of potentially generalizing incomplete disease gene data and overcoming variation in how genes are associated with specific disease terms, improving our ability to detect novel and interesting connections.

We note that a similar principle - that of pooling many weak signals to provide a stronger one - has led to the creation of many highly effective “gene-set analysis” methods for expression data [7], [8] and genome wide association data [9]. However, these approaches are inappropriate for assessing the overlap of disease-linked genes with genes involved in developmental pathways, because the members of our developmental gene sets cannot meaningfully be ranked by the strength of their participation in the set. Standard statistical enrichment methods such as the hypergeometric distribution might be more suitable, but their probabilities depend on inappropriate assumptions of gene independence [10]. Our approach avoids these problems.

The choice of a disease taxonomy for this analysis is vitally important, yet most existing hierarchies lack the molecular focus inherent in the proposed analysis [11]. We chose the MeSH hierarchy of diseases (category C) because it is widely used, it is relatively compatible with our disease-gene databases, and it represents diseases multiple times within different parts of the tree, thus potentially including somewhat molecularly homogeneous groupings [12]. For example, type 1 diabetes mellitus appears multiple times in the taxonomy under categories corresponding to nutritional and metabolic diseases, endocrine disorders, and immune system diseases. The MeSH disease taxonomy can be represented as a “forest” of disease terms (a collection of “trees,” in the computational sense [13]), with 26 top-level categories (Table S1) represented by “disease trees,” and more specific disease terms located at increased tree depths.

We derive our disease-gene links from two sources: OMIM, a curated collection of genes linked to human disease [14], and the Genopedia data from the database of Human Genetic Epidemiology (HuGE), whose disease-gene information is obtained primarily by computational literature curation, but includes manual review of both abstracts and index terms [15]. We then pool genes linked to descendants of a disease node in the MeSH trees, and we assess significance through permutation. Because of the current incomplete knowledge of gene-disease connections, enrichment of gene sets among genes linked to a specific disease node in the MeSH forest may not be detectable. By pooling gene links from related diseases, we are able to rescue some of these lost connections.

For this study, we focus on identifying connections to genes involved in developmental processes. The gene sets chosen were based on Biological Process terms from the Gene Ontology (GO), a hierarchically-organized collection of controlled-vocabulary functional annotation of genes and gene products [16]. However, given our interest specifically in developmental gene sets, we chose to use the gene sets from DFLAT, a prior collaboration of ours that aimed to expand human developmental annotation in the Gene Ontology framework [17]. Gene sets derived from the Gene Ontology that include the DFLAT annotation have been shown to improve the interpretability of gene expression data related to human development [18], so they are a reasonable choice for the analysis described here. We refer to the developmental gene sets whose links to disease are being investigated as the *query* gene sets.

Additional related work assesses significant enrichment of GO functional annotation terms in query gene sets using the directed-acyclic graph structure of the Gene Ontology. Such approaches adjust enrichment calculations by accounting for relationships between the genes at a given annotation node and those at the parent or child [19], [20]. But these methods are concerned with a different problem - that of spurious enrichment at higher levels of the GO hierarchy. Instead, the hazard in our case is false negatives that occur because of the incomplete knowledge of disease genes and the variable levels of precision used to map known disease genes to the MeSH forest. We therefore focus here on query sets representing top-level developmental processes (e.g., “heart development” rather than “atrial cardiac muscle cell development”), because highly specific terms typically include very few genes, rendering gene-set analyses powerless. Future efforts will include drilling down into specific developmental pathways. Yet even at this high level, our analysis identifies both expected links and several unexpected ones, the latter leading to individual novel hypotheses about surprising molecular connections that may affect future disease research.

## Results/Discussion

### A new approach linking gene sets and disease classes

**Figure 1:**
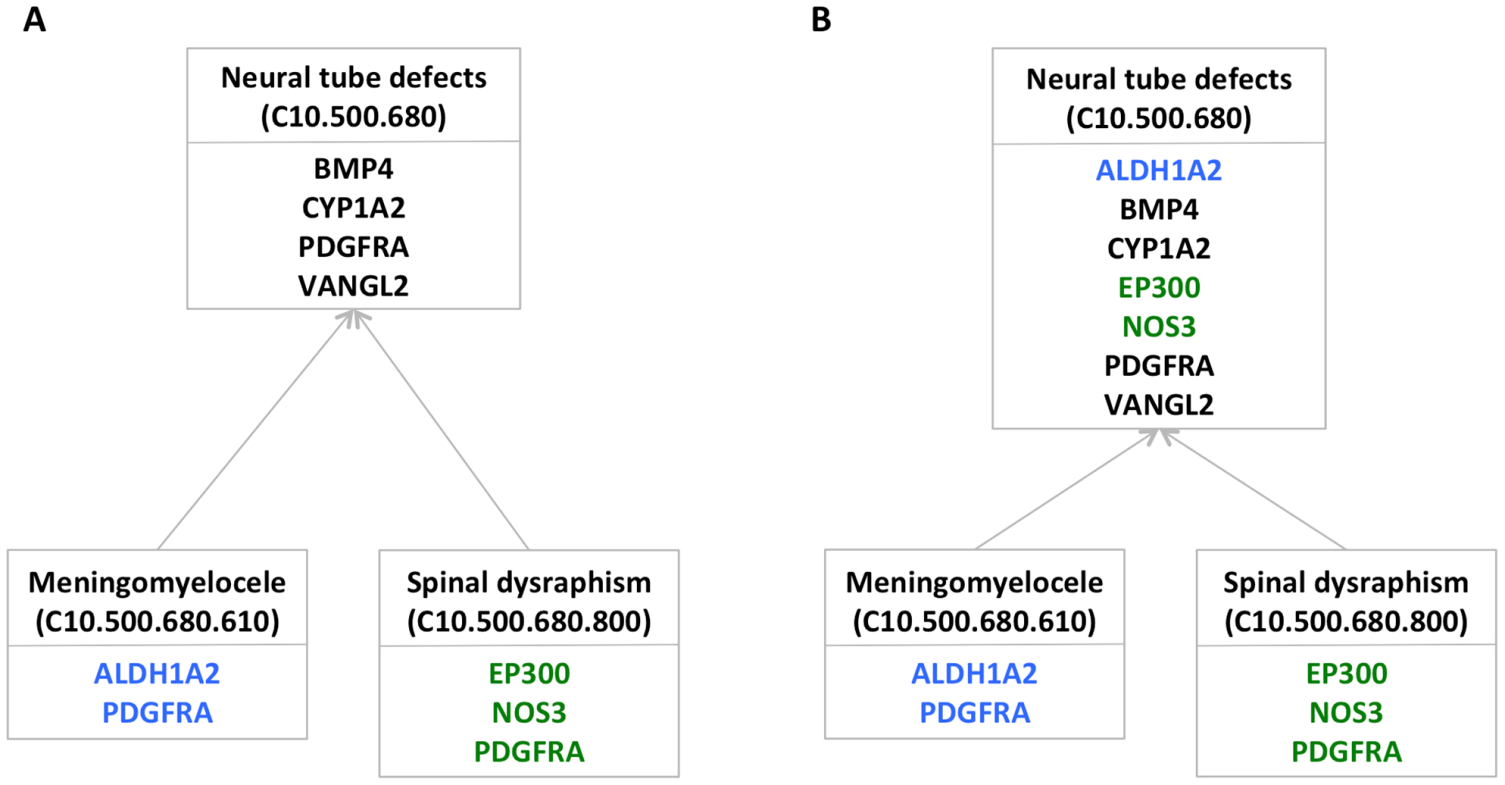
Pooling genes across related diseases to assess enrichment. a) Lung development genes linked directly to three related MeSH terms. The genes associated with each term are shown in a different color. b) By pooling the lung development genes from the subtree rooted at the *Neural tube defects* node, we obtain enough genes to identify significant enrichment at that node. Colors, the same as those in part a, indicate the disease terms with which the genes were associated before pooling.

To identify significant connections between gene sets and disease, we used a novel method of assessing overlaps between disease genes and the designated query gene sets. We first created a computational representation of the MeSH disease taxonomy in which each node represents a MeSH disease concept. We extracted and combined gene-disease links from the HuGE Genopedia database and from OMIM, and mapped the resulting 119,400 gene-disease links to the MeSH forest (see Methods). Taking advantage of the hierarchical representation of disease concepts in MeSH, we then created a version of the forest in which each disease node D contains any genes in the subtree rooted at D. For example, instead of identifying four lung development genes linked to neural tube defects, two to meningomyelocele, and three to spinal dysraphism, pooling them together identifies seven distinct lung development genes implicated in neural tube defects (Figure 1).

For this study we considered nine DFLAT gene sets, broadly representing development in brain, bone, heart, kidney, liver, lung, nerve, blood vessels, and skin. We identified the overlaps between each of these gene sets and the disease genes at each node of our MeSH tree by counting the number of genes in both. (Table S2 lists the query gene sets and their sizes.) Assessing the significance of these overlaps must account for gene set sizes and multiple testing. However, such adjustment is non-trivial because of the complex dependencies between the tests. (For example, any method that assumes the probability of enrichment at node D is independent of the probability of enrichment at D's parent or child is going to be wildly inaccurate.) We therefore use a permutation test (described in the Methods section) to assess the significance of each observed overlap, given the number of genes in the query set and the disease-gene mappings in the MeSH forest. This test produces a p-value at each node estimating the probability of seeing an overlap of the observed size at that node by chance.

### Pooling genes from disease subtrees improves accuracy

Our hypothesis was that mapping disease genes to broader disease terms in the MeSH tree as described above would improve our power to detect actual enrichment by mitigating the effects of varying precision in gene annotation. However, it is also possible that pooling might lead to less-accurate results by incorrectly mapping genes to unrelated disease classes. Assessing which happens more frequently is challenging because the right answers are rarely known. Thus, to compare our pooling approach to a more traditional enrichment analysis, we performed the following experiment.

The intuition behind this experiment is that disease classes that are *correctly* linked to the query gene set should be more likely to be supported by withheld data from the same query set. So we use support by withheld data as a rough way to approximate correctness. Our “pooling” approach computes the significance of the query gene set's enrichment at disease node D by pooling data from the genes in the subtree rooted at D. For fairness, we chose (as the “traditional” method) to assess significance of linkage using exactly the same random permutations of gene labels, but counting only the genes *directly* linked to disease node D (rather than those linked to the node or any of its descendants).

We note that the traditional method used here is really just a randomized approximation to the classical hypergeometric calculation, but one that maintains the correlation structure of genes between different diseases. We have separately computed the hypergeometric probabilities (data not shown), and found them to give very similar overall results to those derived using permutation. Accordingly, we present just the permutation-based method, which is the most direct control for our pooling approach, in the comparison below.

We withheld 100 randomly chosen links, each connecting a gene in the query gene set to a specific associated disease. We recomputed enrichment at each disease node without the withheld links, using both the pooling method and the traditional one. Counting then allows us to estimate the probability 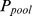 that a randomly-chosen node found to be more significant under the pooling approach than the traditional approach would be supported by a randomly withheld link, and 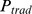, the probability that a node more significant by the traditional method would be. (See Methods for further details.)

**Figure 2:**
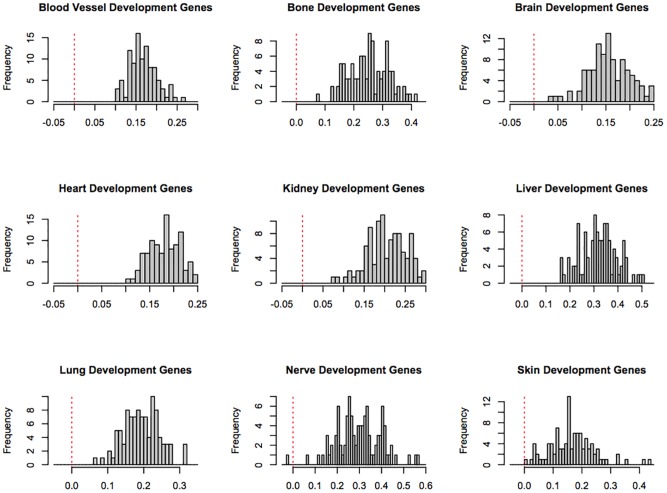
Histogram showing 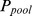 - 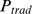 for each query gene set. The red lines show a difference of zero; values to the left of these lines represent individual random trials in which the traditional method outperformed the pooling method. This occurred only once, in one trial for the skin development gene set.

We repeated this experiment with a different set of 100 withheld links 100 times for each of the 9 developmental gene sets. Table 1 shows the average values of 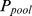 and 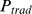 for each of the development gene sets, and Figure 2 shows histograms of the distribution of 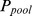 - 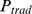 for all of the development gene sets. If 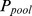 is larger than 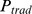, then the nodes that are more significant under the pooling approach tend to be more consistently supported by the withheld data, which is our proxy for correctness. In other words, when 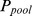 is larger, it suggests that the pooling method tends to make correct links appear more significant. For all nine query sets, we found that the averaged 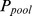 is greater than the averaged 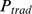, suggesting that the pooling method is better able to identify true links between developmental gene sets and disease.

**Table 1:** Advantage of the pooling approach.

Query Gene Set	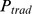	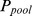
Blood Vessel Development Gene Set	0.0698	0.2428
Bone Development Gene Set	0.1930	0.4574
Brain Development Gene Set	0.1252	0.2887
Heart Development Gene Set	0.0990	0.2781
Kidney Development Gene Set	0.1532	0.3507
Liver Development Gene Set	0.2350	0.5632
Lung Development Gene Set	0.1438	0.3460
Nerve Development Gene Set	0.3296	0.6140
Skin Development Gene Set	0.3007	0.5176

Average probabilities (over 100 trials) that random, withheld gene-disease links support nodes more significant by the traditional method (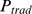) or the proposed pooling method (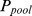) for the 9 query gene sets. Significance in each trial was computed without the withheld links. When 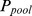 is larger than 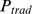, the nodes that are more significant under the pooling approach tend to be more consistently supported by the withheld data, our proxy for correctness.

### A visualization tool for connecting gene sets and disease

While it is relatively easy to provide a list, for each developmental gene set, of MeSH terms whose gene set enrichment p-value is below some cutoff, interpreting those lists is complex. Because enrichment calculations are based on subtrees, there is important information available at different scales, ranging from high-level overviews of the MeSH disease forest to specific enriched gene-disease links, their significance scores, and the genes involved. For these results to lead to new discoveries, we must select from this large collection of significant links a few that are surprising yet plausible. Doing this requires a considerable amount of domain knowledge in molecular medicine.

**Figure 3:**
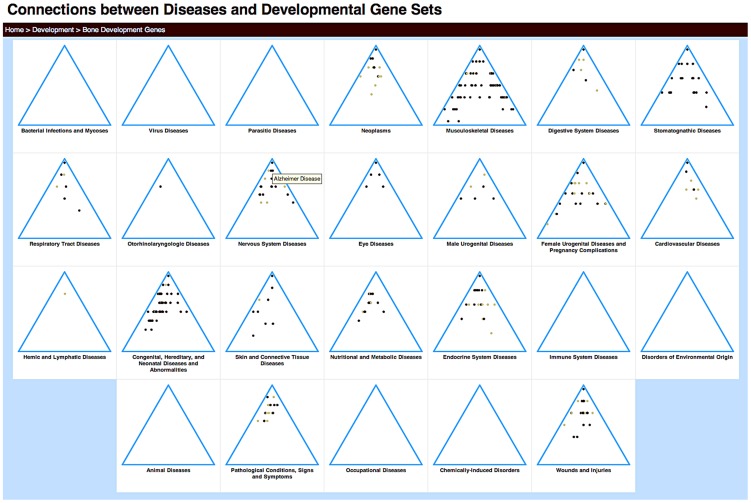
Triangle view of disease enrichment for the bone development gene set. Each triangle represents one of the 26 top-level categories in the MeSH disease forest. Each dot represents a disease node with significant enrichment of brain development genes. To clearly indicate the significance of relationships between diseases and the query gene set in these small images, we used two colors: light brown dots indicate 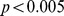, and darker brown dots, 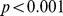. Mousing over the dots reveals a pop-up of the disease term associated with that node (Alzheimer Disease is shown). Clicking on the category name leads to a detailed view of that tree.

To facilitate data exploration by collaborators with such expertise, we developed a web-based tool that provides both an abstract and a detailed view of the associations (available at http://gda.cs.tufts.edu/development). For a high-level overview, we visualize each disjoint hierarchy of disease terms (i.e., each tree of the MeSH disease forest) in a simplified triangular form (Figure 3). Each significant disease association with the given gene set is represented as a dot in this triangle, whose color represents the degree of significance. This abstract view helps highlight the broad overall patterns of association between development gene sets and disease classes.

**Figure 4:**
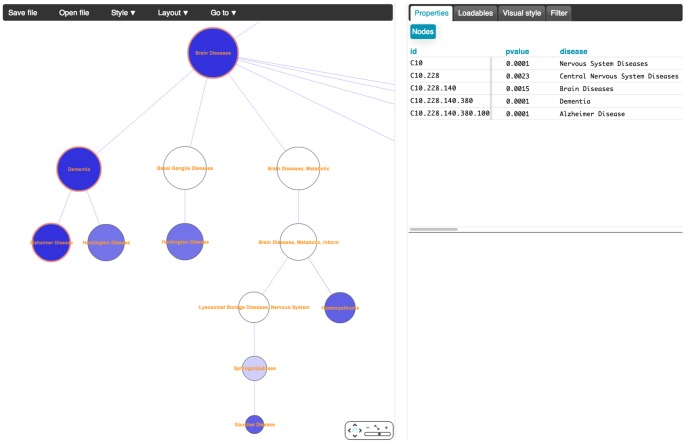
Detailed view of part of the Nervous System Disease subtree, showing enrichment of bone development genes. Links to dementia and Alzheimer's disease are shown. Significance of each node in the tree is represented by color; a gradient of shades of blue indicates p-values ranging from 0 (darkest blue) to 1.0 (white). Clicking on a node or selecting a set of nodes allows users to see, in the box in the upper right corner, the selected disease terms, p-values, and genes shared between those diseases and the developmental gene set.

Clicking on a particular disease subtree leads to a detailed tree view (Figure 4). The tree visualization is implemented using Cytoscape Web [21]. Color again corresponds to significance, with darker nodes indicating more significant enrichment of the developmental gene set in the disease genes associated with the subtree rooted at that node. For clarity, this view by default only displays disease nodes significantly associated with the query gene set (and their ancestors in the chosen tree). However, users can adjust parameters to view the full tree if desired. Specific genes and p-values for individual links can be identified by selecting nodes in this view. The associated gene lists are easily selected and pasted into functional analysis tools for pathway identification.

In the next two sections, we describe some results from our initial explorations using this tool. The first section provides a sanity-check by demonstrating that we find the broad patterns of connections that one would expect, while the next shows that we can use this approach and the tool described here to make novel but plausible discoveries with potential clinical impact.

### Developmental gene sets implicated in expected disease trees

**Figure 5:**
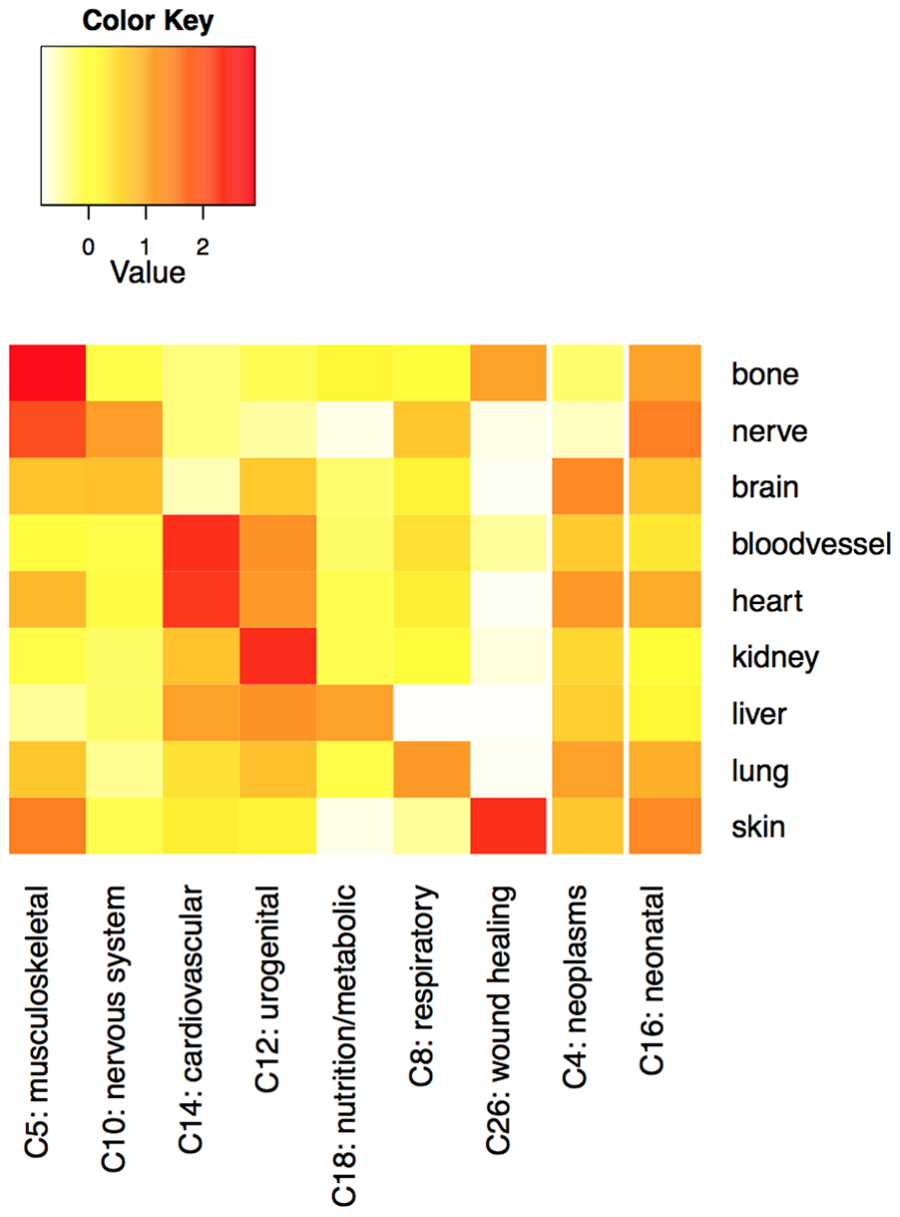
Expected results by tissue. Density of enrichment of developmental gene sets (labels on the right) in major disease subtrees. Values are z-score normalized densities, computed as described in Methods. Darker squares indicate that a larger fraction of the disease terms in the MeSH category have significant enrichment (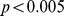) of genes in the indicated gene set. Expected connections appear approximately along the diagonal in the first 7 columns, and throughout the rightmost two columns.

We first take a high-level view of all the results together. Generally speaking, one would expect to see connections between tissue-specific developmental gene sets and broad categories of diseases known to involve those particular tissues. For example, it seems likely that many cardiovascular disorders would be linked to a significant number of heart development genes. Figure 5 shows a heatmap of the relative “density” of disease terms significantly linked to each of the gene sets (see Methods) for several MeSH disease trees. We see high enrichment that essentially mirrors our expectations: bone development genes are over-represented in musculoskeletal disorders, brain development genes in nervous system disorders, heart development genes in cardiovascular disorders, etc.

There are a few interesting exceptions. For example, the percentage of nervous system disorders significantly enriched for nerve development genes is relatively high, but not quite high as the percentage of musculoskeletal diseases enriched for nerve development genes. This seems to be in part an artifact of the large number of distinct nervous system disorders listed in MeSH category C despite having little or no molecular information, artificially decreasing the normalized density values (the maximum density score in the C10 category is lower than the maximum score in any of the other MeSH disease trees shown in the figure).

The root node of MeSH category C4, “Neoplasms,” is significantly associated (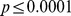) with all of the developmental gene sets except for nerve and skin (the two smallest of the gene sets and therefore the least likely to have significant overlaps). This observation reflects the fact that the regulation of cell growth and differentiation that comprise normal developmental processes are typically disrupted and dysregulated during the onset of malignancy [22], [23]. A range of signaling proteins that play roles in directing both developmental processes and tumorigenesis are likely to blame for these interactions [24]–[26]. However, the specific signaling processes implicated in the different tumor types, as well as those known to be involved in developmental processes but not yet implicated in specific tumor types, may be of interest.

Similarly, given that the query gene sets are all involved in developmental processes, it is not surprising that the C16 MeSH subtree, described as “Congenital, Hereditary, and Neonatal Diseases and Abnormalities,” shows significant enrichment at the root node (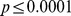) for all of the tested developmental gene sets. A wide range of molecular developmental processes are implicated in this MeSH category. The density measurement shown in Figure 5 provides a broader way of assessing a similar property. The density measure for the C16 tree is above average (i.e., the z-score normalized density metric is positive) for each of the nine gene sets considered here.

By confirming that we find expected and reasonable high-level results, the observations in this section provide evidence of the efficacy of our approach.

### Unexpected connections and implications

Delving more closely into specific results, we identified several findings that seemed, at first glance, less predictable than those described above. Here we describe three such links. All of them identified surprising connections that, since our initial discovery of them using this approach, have been further supported by new publications.

#### Bone development and dementia

One surprising link is a significant overlap (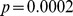) between bone development genes and genes involved in dementia (MeSH term C10.228.140.380). There are 24 genes involved in this overlap. One might suspect that the connection would be through BMP signaling proteins, which play developmental roles in a variety of processes including bone formation and neurogenesis. Yet although *BMP4* is among the 24 genes, it is the only BMP family member on the list. Functional analysis (in DAVID [27], v6.7) of the linking gene set indicates enrichment of a broader set of proteins involved in bone morphogenesis (*COL1A1, COL13A1, HSPG2, PEX7*, and *RUNX2*). This is to be expected in a subset of genes involved in bone development. Yet we also saw enrichment of retinoic acid receptor proteins (*RARA, RARB, RARG*) and heparin-binding proteins (*BMP4, COMP, COL13A1, FGFR2*). These links are of interest because both heparin derivatives and retinoic acid are candidates for new Alzheimers therapies [28], [29], yet both are also known to contribute to osteoporosis [30], [31].

Evidence supporting this connection has recently been proposed in empirical observations of an association between lower bone mineral density and dementia in postmenopausal women [32]. Although molecular pathways supporting this link were not identified, a role for estrogen deficiency was suggested. Our observations are consistent with this hypothesis – five of the 24 shared genes (*ALPL, BMP4, COL1A1, GH1*,and *RARA*) are among those with the GO Biological Process annotation “response to steroid hormone stimulus,” a finding whose adjusted false discovery rate (as computed in DAVID via the Benjamini-Hochberg method) is below 0.015.

This analysis also suggests a possible connection between dementia and bone density through additional signaling pathways. For example, the growth factor *MDK*, a still relatively unstudied, retinoic acid-responsive, heparin-binding protein appears to be involved in both neuron and bone growth [33]. Elevated levels have been observed in serum from Alzheimer's patients [34]. Our observations suggest that molecular connections through this and related signaling pathways may be worth exploring in the quest for novel therapeutic approaches to dementia.

#### Heart development and polycystic ovary syndrome

The link between heart development and polycystic ovary syndrome (PCOS; MESH term C19.391.630.580.765) has a p-value below 0.0001. PCOS is an endocrine disorder that causes hormonal changes, ovarian “cysts” (that are actually immature follicles), and decreased female fertility. It has been associated with an increased risk of diabetes, dyslipidemia, and cardiovascular disease [35]. There are 31 genes responsible for the connection we observed between PCOS and heart development. Functional analysis of this gene list shows enrichment of genes annotated with the GO Molecular Function term “SMAD binding” and those in the KEGG “TGF-beta signaling” pathway. TGF-beta (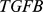) is the canonical member of a family of cytokines that play regulatory roles in many developmental, homeostatic, and immune processes. It regulates apoptotic pathways, in part through SMAD binding [36].

It has long been known that cardiovascular symptoms are associated with PCOS, but the molecular etiology of this connection is not clear. One study proposed that oxidative stress caused by insulin resistance may lead to cardiovascular injury in nonobese PCOS patients, but did not implicate specific molecular pathways [37]. Given that *TGFB/SMAD* complexes are known to mediate the DNA damage response [38], [39], dysregulation of 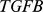 is a possible mechanism to be considered.

A role for 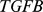 in PCOS through mutations in fibrillin 3, a gene linked to PCOS, has also recently been suggested. Fibrillin 3 expression changes in fetal ovaries of PCOS patients have been shown to affect 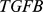 binding, perhaps leading to changes in follicle formation [40]. The same paper suggested that the PCOS phenotype was consistent with increased 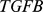 activity. A more direct role for 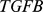 itself in PCOS was recently proposed, despite largely circumstantial evidence [41]. Our analysis also seems to support this hypothesis, which potentially explains both the observed cardiovascular outcomes and the early developmental origins of ovarian cysts in PCOS.

We looked for further corroborating evidence in mouse models, but could not identify an existing, well-characterized mutant that is a good model of 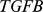 *upregulation*. However, there are four genes in the KEGG TGFb pathway that are known to inhibit 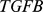 activity: *LTBP1, DCN, Lefty*, and *Activin*. For all of these genes there are mouse mutant strains (in a variety of backgrounds) that disrupt the homologous proteins' expression, thus potentially upregulating 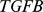. These mutant strains have differing degrees of phenotypic characterization, but two of them (*DCN* and *Activin*) have knockout mutations that cause reduced female fertility [42], [43], and the activin knockouts are even characterized as having ovarian cysts. These findings are unexpected by chance: the hypergeometric probability of seeing at least two of four randomly-selected proteins whose knockout strains are characterized as having reduced female fertility in the Mouse Genome Database is below 0.0001, as is the probability of seeing at least one of four with an ovarian cyst phenotype. We therefore suggest that further work on the role of the 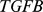 pathway in the development of PCOS may prove fruitful.

#### Lung development and retinopathy of prematurity

The lung development gene set was linked, with a p-value below 0.0001, to retinopathy of prematurity (MeSH term C16.614.521.731). Retinopathy of prematurity (ROP) is a complication that occurs primarily in infants delivered before approximately 28 weeks' gestational age, before the infants' visual system has been fully formed [44]. While early detection and treatment often lead to a full recovery, severe cases may lead to permanent nearsightedness or vision loss [45]. Yet we still know too little about why some infants develop this complication of prematurity, while others born at the same age and with similar clinical characteristics do not. Although most neonates with retinopathy of prematurity also have immature lungs, a molecular connection between ROP and lung development is not readily apparent.

The significant connection we observed was based on five genes linked to both ROP and lung development: *IGF1, NOS3, EPAS1, KDR*, and *VEGFA*. These genes are all related to blood vessel or tube development. Excessive but disordered VEGF-mediated vascularization of the retina is known as the cause of ROP [46], and indeed ROP has been successfully treated in recent pilot studies by intravitreal administration of the VEGF inhibitor bevacizumab [47]. It appears likely that these genes may be playing a specific role in alveolar development and lung function.

We therefore hypothesized that there might be similar molecular enrichment of the ROP genes in bronchopulmonary dysplasia (BPD), another complication of prematurity characterized by extended need for supplemental oxygen and, in extreme cases, long-term respiratory insufficiency. Like ROP, BPD also affects some, but not all, extremely premature infants. Its exact cause is unknown. Current hypotheses include one in which inflammation plays a major role [48], as well as the so-called vascular hypothesis of BPD in which decreased vascularization impairs alveolar formation at a critical time [49].

**Figure 6:**
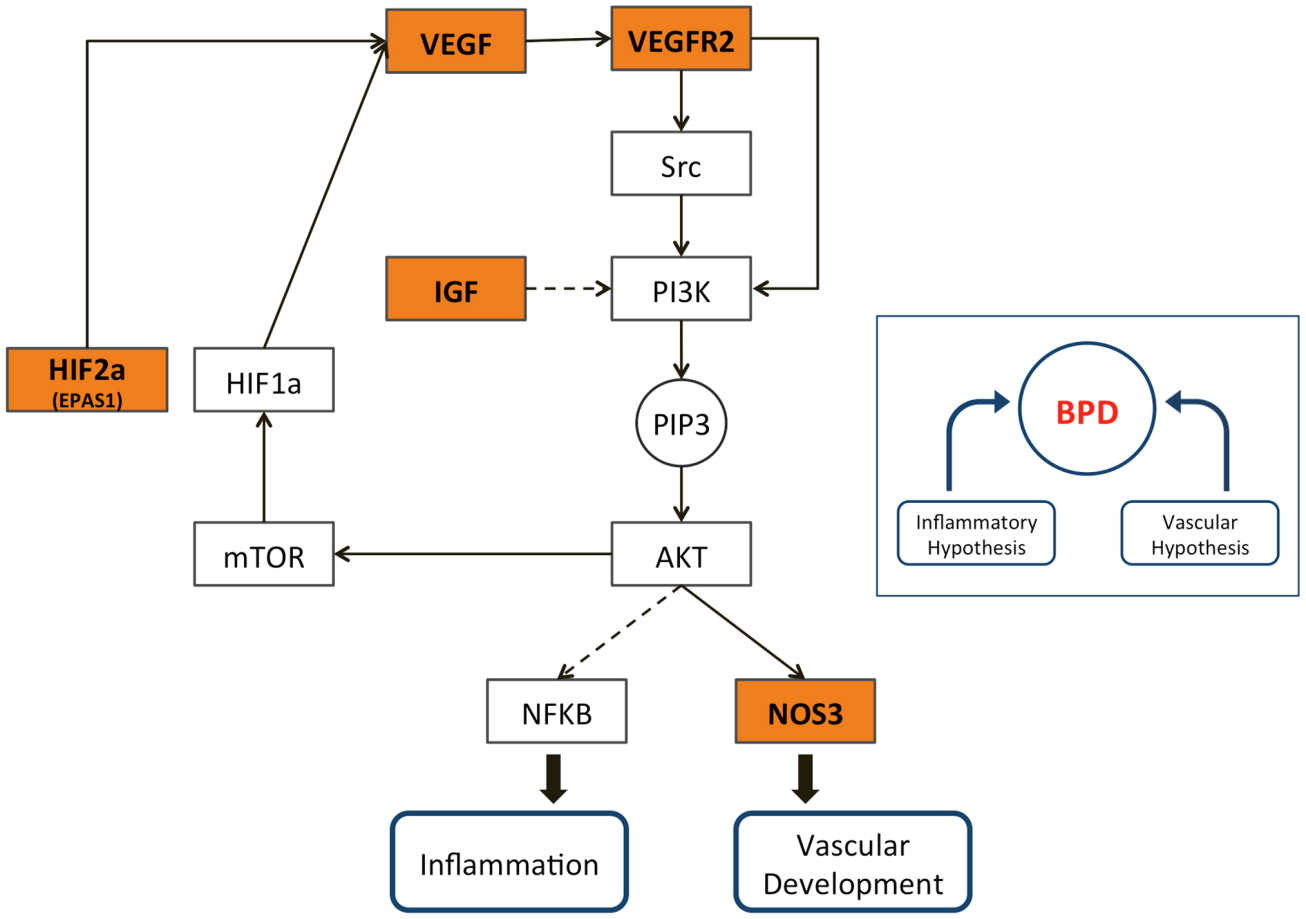
The VEGF pathway and its relevance to both BPD hypotheses. The relationships shown here are derived from the VEGF, PI3K-AKT, mTOR, and HIF-1 signaling pathways and the “Pathways in Cancer” map in the KEGG Pathway database. Dashed lines represent indirect regulation. Genes highlighted in orange are the five lung development genes implicated in ROP.

Our observations are consistent with the hypothesis that both complications may be caused in part by perturbations of the *VEGF* pathway (Figure 6), which provides a molecular link between the vascular and inflammatory BPD hypotheses. In support of this theory, we observe that 7 of the 26 ROP genes are among the 49 BPD genes listed in our disease-gene collection, an overlap that would occur by chance with a hypergeometric probability of 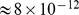. We also wondered whether the two disorders tend to occur in the same infants more often than expected by chance. This question was recently answered in the affirmative, in a paper that did not identify the cause of such clustering but hypothesized that both ROP and BPD might be consequences of NICU-acquired infection [50]. More recent work shows an association between low VEGF protein levels in urine and the eventual development of both ROP and BPD [51], suggesting a non-invasive early approach to predict both outcomes.

However, the picture appears to be more complex than this. If both ROP and BPD were associated with uniformly low VEGF activity, then the current practice of treating ROP with VEGF *inhibitors* presumably would not have arisen. BPD, on the other hand, does appear to be associated with reduced *VEGF* expression levels in the lungs of human neonates [52], and administration of VEGF ameliorates symptoms of respiratory distress in a mouse model of BPD caused by inhibition of the *VEGF* pathway [53]. It is possible that an interaction between VEGF levels and NFKB-mediated response to neonatal infection accounts for the observed co-occurrence of these disorders.

There is also a possible connection to adult pulmonary disease [54]. Drug-induced suppression of VEGF has been used to create a model of emphysema in adult rats [55]. A prior analysis of gene expression in BPD implicated chromatin remodeling and histone acetylation pathways [56]. We therefore investigated possible molecular links between BPD and chronic obstructive pulmonary disease (COPD), in which histone acetylation also plays a role [57], [58]. We observed that 26 of the 49 BPD genes in our data set are among the genes linked to COPD (an event that would occur under the null hypothesis with hypergeometric probability 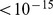). It is difficult to directly assess co-occurrence of BPD and COPD in the same individuals, because COPD typically affects older patients, most of whom were born before the neonatal diagnosis of BPD was formally defined in 1967 [59]. However, the observation of early-onset emphysema (a form of COPD) in BPD survivors [60] supports our theory that this molecular overlap does not occur by chance. Our evidence leads to the hypothesis that there might be a shared molecular mechanism predisposing individuals to an excessive response to alveolar damage, whether the damage is caused by oxidative stress from neonatal ventilation, or cigarette smoke in adults.

### Conclusions and future work

We have introduced a new approach that identifies significant overlap of gene sets with groups of related diseases in a hierarchical disease taxonomy. To evaluate this approach, we implemented a tool that allows users to explore connections between disease subtrees in MeSH and several developmental gene sets. Our observations in this analysis have helped identify surprising molecular connections between disparate processes. They have also more generally served to validate the approach of pooling incomplete information about disease genes across related disorders to strengthen our ability to identify such connections. With a growing interest in research into the developmental origins of adult disease, this resource should prove a valuable source of information for generating hypotheses about such connections at the molecular level.

Our work has assumed only that query gene sets are lists of genes that share some common property [61]. However, for this study we have chosen query sets whose genes share common annotations in the Gene Ontology. An interesting future direction would be to consider the possibility of creating hierarchically-structured queries representing related query terms in the Gene Ontology's directed acyclic graph structure, while still looking for significant links to disease classes or subtrees in the MeSH forest.

While our implementation relies on a particular set of disease-gene information and a small group of developmental gene sets, the power of the approach will be best exploited by the inclusion of a more comprehensive set of disease-associated genes. One key limitation of the current approach is due to the nature of the available data linking genes to diseases. OMIM is an excellent resource created largely by computer-assisted manual review of the literature [14]. However, it is limited in scope and is curated by locus rather than by disease, so that even identifying all genes related to, for example, type 2 diabetes, can be complicated. Conversely, the HuGE database, which provides the majority of the disease-gene data used in this project, derives most of its information from computational screening of PubMed (along with some manual review) [62], [63]. This raises the possibility that, in addition to being incomplete, our gene-disease database may include a substantial number of false positives due not only to false-positive experimental results but also to inappropriate interpretation of the text. There is prior work on reducing the rate of false positives when mining such information from the literature [64], and the HuGE database creators worked to assess and improve accuracy [63], but any data set derived from computational literature analysis will always have this concern. On the other hand, the success of our initial analysis in identifying expected connections suggests that false positives are so far not interfering significantly with the use of this tool for discovery. Further improving the quality of the data and characterizing the impact of different types of noise on the results will be an important area to investigate in the future.

Finally, we note that while there are many disease taxonomies that are widely used for different purposes, there is growing dissatisfaction with most of them, in part because of the lack of a molecular representation of disease relationships [11]. Analyses such as ours may, as the data improve over time, lead to better understanding of molecular disease relationships across the board. Such knowledge is an important prerequisite for developing a truly molecular taxonomy of disease. We therefore hope that this work may ultimately contribute to the development of a new, more molecular disease taxonomy that is well suited to support translational research in the genomic era.

## Methods

### Gene-disease data

We assembled a combined set of disease-gene links for 11,831 genes using 116,117 human gene-disease associations from the Genopedia compendium in the HuGE database of Human Genetic Epidemiology [62] and 4,813 gene-disease associations from the OMIM database [65], both downloaded in November, 2013. Genes from the Genopedia database were mapped to their corresponding disease concepts in the MeSH hierarchy of medical subject headings (http://www.nlm.nih.gov/mesh/), using the Unified Medical Language System (UMLS) [66] as a thesaurus to identify synonymous diseases. To find MeSH terms that best correspond to the OMIM phenotypes, we used the MEDIC merged disease vocabulary, an ongoing toxicogenomics effort to map OMIM disease terms into the MeSH disease hierarchy, downloaded from the Comparative Toxicogenomics Database [67] in November, 2013. After removing one copy of the 1,530 duplicate associations found in both data sets, we were left with a total of 119,400 unique associations.

### Estimating significance

We estimate the distribution of the expected number of shared genes between the query gene set and the genes associated with a disease under the null hypothesis that there is no meaningful relationship between the query gene set and the disease class. We do so by randomly choosing gene sets of the query-set size from among all the genes in our MeSH tree. This is equivalent to randomly permuting the labels of the genes in the data to determine whether or not they are in the query set. Such permutation leaves the gene-disease connections intact and maintains the complex correlation structure of genes between related diseases. Assuming that 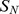 is the observed size of the real overlap at disease node 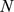 (i.e., the number of genes in the query gene set that are linked to node 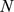), for each permuted query set we can then determine whether the number of genes at node 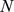 in that random query set is larger than 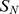. We ran 10,000 permutations to compute a p-value at each node estimating the probability of seeing an overlap of the observed size at that node by chance.

### Density of significant enrichment

Density of enrichment was computed between the 9 query gene sets and the 26 top-level MeSH disease categories, each represented by its own tree. Because many diseases are represented multiple times at different places in each tree, we first created a listing of all the unique MeSH disease terms in each tree. If different instances of the same disease in the same tree had different p-values, they were averaged. We then compared the p-values to the chosen significance cutoff of 0.005. The fraction of unique terms in the tree with lower significance was computed. This fraction represents the “density” of significant enrichment of the query gene set in the chosen MeSH category.

To create the heatmap, we z-score normalized the densities across each row (query gene set). To identify expected enrichment, we manually selected the 9 top-level MeSH disease categories thought to be most relevant to the 9 query gene sets (or to many/all developmental gene sets, as in the case of C4 - neoplasms and C16 - congenital, hereditary, and neonatal diseases and disorders).

### Comparing the accuracy of the pooling and traditional approaches

We performed the following experiment to compare the accuracy of our proposed pooling approach to a comparable enrichment analysis using only the genes directly associated with a given disease term. To describe the experiment, we first introduce new terminology:

Assume that we are discussing only a single, fixed query gene set. Let 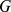 be the set of all gene-disease links in our combined database: 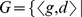 gene 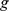 is associated with disease 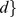. For any disease node 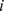 in the MeSH forest, let 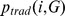 be the permutation-based significance score for enrichment of the query gene set among genes in 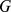 associated with that node using the traditional method (only those genes directly linked to node 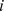). Similarly, let 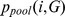 be the analogous score for node 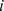 under the pooling approach.

Then we will repeatedly randomly withhold some links from 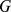. Specifically, for the 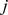
th random iteration, let 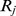 be a randomly chosen set of 100 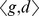 pairs from 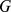, such that 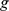 is in the query gene set, and let 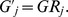 We can then partition the disease nodes into those that are more significant under the pooling method (in the 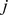
th iteration) and those that are more significant under the traditional method. Formally, let 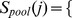 nodes 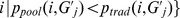, and let 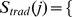 nodes 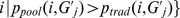. (Note that in the many cases where 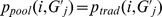, the nodes contribute to neither set. Many of these are either leaves, or nodes with no associated genes under either method.)

**Figure 7:**
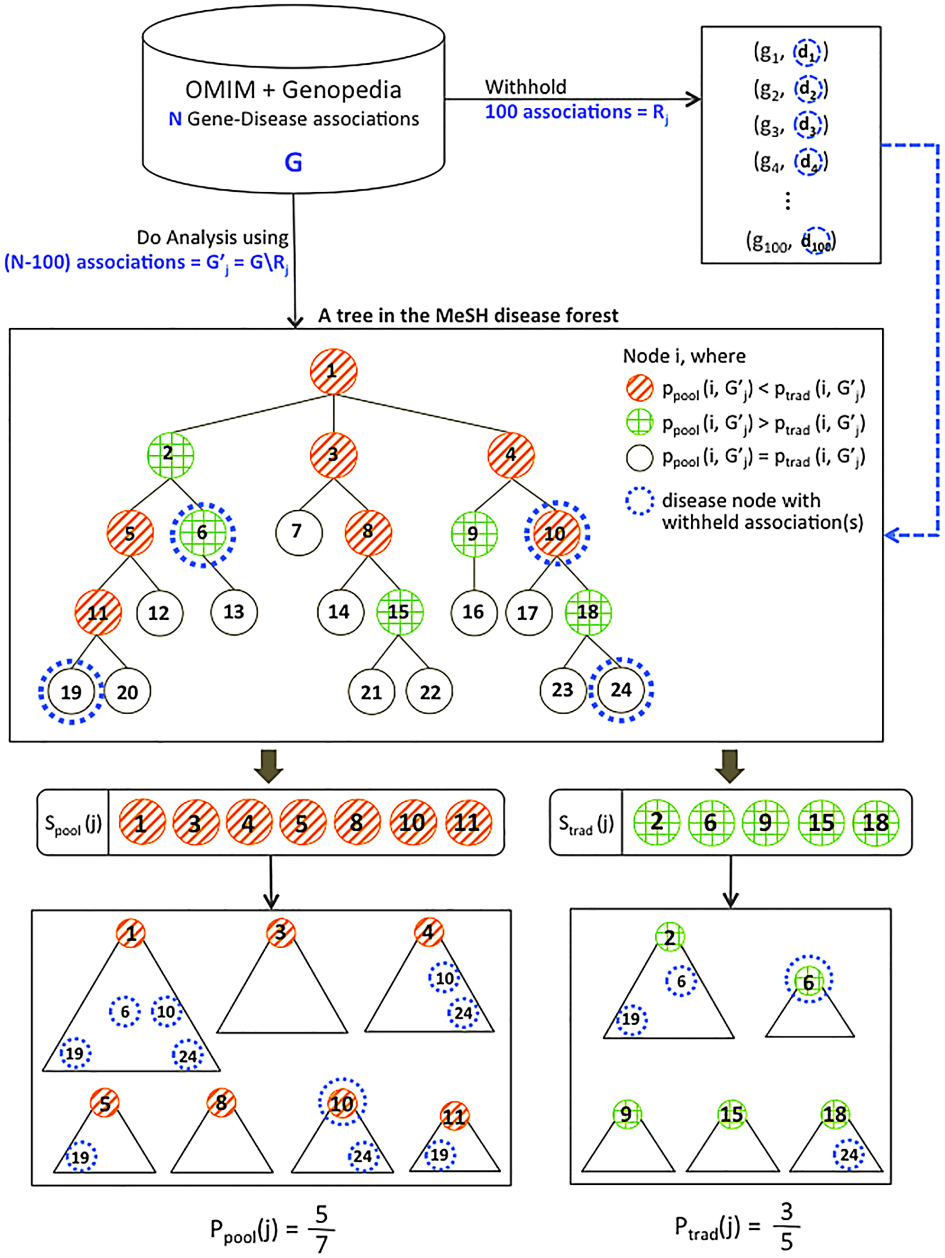
Example of comparison between pooling approach and traditional approach. Illustration of the process for calculating 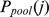 and 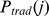 for the 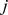
th random trial. 100 gene-disease associations involving genes in the query gene set are withheld. Using the remaining associations, p-values for enrichment of the disease gene set at each node are computed using both the traditional and pooling approaches. Nodes are assigned to 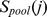 or 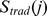 based on which approach shows more significant enrichment, and the rate at which each set is supported by withheld links is computed. The idea is that if a disease class is correctly linked to the query gene set, it should be more likely to be supported by withheld gene-disease associations from that same query set.

We say a node 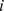 is *supported* by gene-disease link 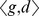 from 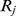 if a node corresponding to 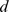 appears in the subtree rooted at 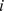. We can then determine the probability that a node in the set 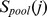 or 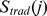 is supported by some link in 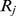. Let indicator function 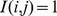 if node 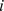 is supported by a link in 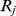, and 0 otherwise. Then the probability that a node in 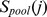 is supported by 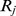 is defined as
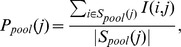
and 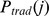 is defined analogously, using 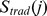 Finally, we average over all random trials 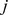 to compute the averages 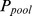 and 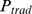 that are reported in Table 1. Figure 7 illustrates the process of calculating 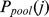 and 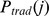 with an example for the 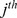 random trial.

## Supporting Information

Table S1 **List of 26 top-level categories in the MeSH disease (C) forest.**
(PDF)

Table S2 **The 9 query gene sets and their sizes.**
(XLSX)
