# Supplementary material for: Finding Novel Molecular Connections between Developmental Processes and Disease
Source: PLoS Comput Biol. 2014 May 29;10(5):e1003578. doi: 10.1371/journal.pcbi.1003578 (PMC4038461; doi:10.1371/journal.pcbi.1003578)
Supplement: Table S1 — List of 26 top-level categories in the MeSH disease (C) forest. (PDF) [file pcbi.1003578.s001.pdf]

List of 26 top-level categories in the MeSH disease (C) forest

MeSH Index	Disease Name
C01	Bacterial Infections and Mycoses
C02	Virus Diseases
C03	Parasitic Diseases
C04	Neoplasms
C05	Musculoskeletal Diseases
C06	Digestive System Diseases
C07	Stomatognathic Diseases
C08	Respiratory Tract Diseases
C09	Otorhinolaryngologic Diseases
C10	Nervous System Diseases
C11	Eye Diseases
C12	Male Urogenital Diseases
C13	Female Urogenital Diseases and Pregnancy Complications
C14	Cardiovascular Diseases
C15	Hemic and Lymphatic Diseases
C16	Congenital, Hereditary, and Neonatal Diseases and Abnormalities
C17	Skin and Connective Tissue Diseases
C18	Nutritional and Metabolic Diseases
C19	Endocrine System Diseases
C20	Immune System Diseases
C21	Disorders of Environmental Origin
C22	Animal Diseases
C23	Pathological Conditions, Signs and Symptoms
C24	Occupational Diseases
C25	Chemically-Induced Disorders
C26	Wounds and Injuries
